# A step-by-step guide for mentors to facilitate team building and communication in virtual teams

**DOI:** 10.1080/10872981.2022.2094529

**Published:** 2022-06-28

**Authors:** Julia F Aquino, Robert R Riss, Sara M Multerer, Leora N Mogilner, Teri L Turner

**Affiliations:** a Department of Pediatrics, Boston Children’s Hospital, Boston, MA, USA; b Department of Pediatrics, Children’s Mercy Hospital, University of Missouri at Kansas City, Kansas City, KS, USA; c Department of Pediatrics, Norton Children’s Medical Group affiliated with University of Louisville, Louisville, KY, USA; d Department of Pediatrics, Icahn School of Medicine at Mount Sinai, New York, NY, USA; e Department of Pediatrics, Baylor College of Medicine and Texas Children’s Hospital, Houston, TX, USA

**Keywords:** Distance mentoring, communication, team building, shared leadership, virtual teams

## Abstract

As collaborative work in medical education has increasingly moved online, team mentors have had to adapt their practices into the virtual environment. Fostering connection, communication and productivity on virtual teams requires specific skills and deliberate practice that differ from in-person teamwork. Drawing from best practices in business, education and medicine and also from our own experience as a virtual team, we present a guide for mentors to create and sustain successful virtual teams. Grounded in Tuckman’s Five Stage Model of Team Development, we offer specific strategies for virtual team mentors to promote team cohesion, mitigate conflict, maintain productivity and leverage the benefits of the virtual environment.

Our world continues to grow smaller with newer modes of technology that can facilitate connections among team members. In March 2020, teams found it imperative to migrate their activities to a virtual environment, and mentors, or those individuals most experienced on the team typically charged with providing guidance, were called upon to help make this happen. What was once considered a temporary inconvenience has now become a way of life. In a world that requires distancing between colleagues, whether across the hall or across the country, teams must meet the challenge of communicating, team building, and mentoring.

Virtual teamwork presents both challenges and opportunities not faced when collaborating in-person. The aim of this article is to present specific strategies that mentors of virtual teams can use to facilitate team building and improve communication. Our strategies are organized around the Five Stage Model of Team Development by Bruce Tuckman, which includes Forming, Storming, Norming, Performing and Adjourning (Figure 1)[1]. We have specifically chosen to focus on guiding principles rather than technology, because technology, although a useful tool to achieve a goal, does not define the goal itself. In addition, while technology changes quickly, the principles behind team building remain consistent. This article is a synthesis of our own experiences as a virtual team, integrated with knowledge and insights from the fields of sociology, business, technology, and higher education. We have applied many leadership concepts from these disciplines to develop strategies and tools for group mentorship and collaboration. These tips are relevant to fully virtual as well as hybrid teams and teams composed of students, faculty or those with mixed levels of learners. We hope that this information will assist other teams and their mentors navigate the virtual world more effectively and efficiently.

**Figure 1.**
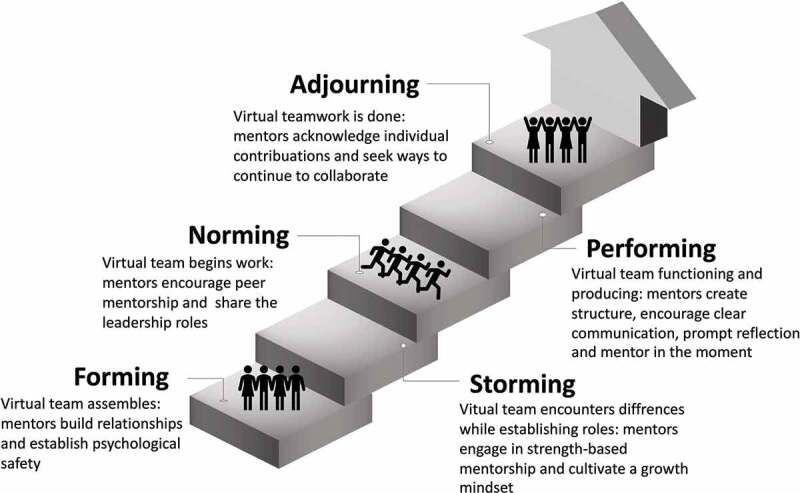
Five stage of team development by Bruce Tuckman.

## Stage 1: forming

In this first stage of virtual team development, individuals assemble and a mentor must have strategies to cultivate cohesion and positive team dynamics.

### Build relationships at the outset

Successful group work requires a commitment to a shared purpose, as well as initial bonding and establishing connections[2]. Early relationship building fosters trust, which enables cohesion, commitment, and psychological safety in a team of workers[3]. For group work at a distance, like in-person group work, team success depends on developing strong relationships, trust and a shared mental model of the direction of the team. This task is more challenging when collaborators span different institutions and lack everyday opportunities to get to know one another. Experts in online student education have established three best practices for developing virtual and online communities: initial bonding, support of continued interaction, and maintaining multiple avenues for communication[4].

What are some strategies for promoting bonding and connections when collaborating across a distance? Consider beginning a new project with a welcome call designed to ‘break the ice’ and get to know your collaborators. With each follow up call, resist the urge to dive right into work. Preserve time on the agenda to socialize and make personal connections. Trust develops more slowly in groups that work solely on virtual platforms[5]. If you are not communicating using videoconference, try to find opportunities for face-to-face connection. If you are going to be in the same place as your collaborators, schedule a time to meet, work, or chat. Meeting in person when possible, even with only one colleague from a larger group, can be encouraging and energizing.

### Create psychological safety

Psychological safety in an environment where individuals feel comfortable expressing themselves and taking risks is essential to successful team functioning. [6,7] But this sense of security may be more difficult to establish in virtual teams, where individuals may have never met before and socializing outside of the work setting is not possible. A virtual team mentor needs to purposefully create psychological safety for the team within the constructs of the online environment. It helps when team members can see each other, so encourage all team members to turn on their cameras during virtual meetings. Discourage faceless multi-tasking during virtual meetings[8]. Inclusive body language and eye contact are difficult in the virtual environment, so the mentor should refer to team members by name. To set a lighter tone for the group, smile, promote laughter, and even consider changing your voice to a higher pitch[9]. Virtual teams can create rituals that promote psychological safety, such as starting with a ‘round table’ sharing activity so the voices of all members – even the quieter ones – are heard from the outset. One strategy to promote psychological safety is for mentors to share stories with the team of their own professional (or personal) challenges or missteps, to promote more risk taking.

## Stage 2: storming

In this next stage of virtual team development, individuals may experience conflict while defining roles on the team. Here, we focus on the mentor’s role in anticipating and mitigating conflict in order to foster team collaboration.

### Engage in strength-based mentoring

Team building can often start with a process of sorting out the differences between team members. The most effective teams are structured to allow individuals to capitalize on their strengths, and also welcome the strengths of others. A team needs to be cohesive while encouraging the diversity of each member’s contribution. Large Gallup surveys have demonstrated that employee engagement is about 65% higher for those who feel their strengths are appropriately used compared to those who do not[10]. Getting to know the strengths of team members is especially important for a virtual team, so the mentor can appropriately assign roles and responsibilities[8]. A variety of online tools included in the references are available commercially to help identify which team member might be best for which role [11–13]. A team that embraces the strengths of all members will be more well-rounded and effective.

Recognizing individual strengths not only helps a team function efficiently, but can also help mentors provide one-on-one coaching in the context of group work. Individual mentoring is particularly important in the storming phase of virtual team formation, where team members may be vying for roles and attention, and mentors need to take steps if competition is becoming a barrier to team functioning. Mentors may need to make themselves available outside of group meetings for one-on-one meetings. By being sensitive to the needs of individual team members, a mentor can coach and support each team member within the context of the larger team goals. Mentors should explicitly acknowledge unique team member contributions, while always ensuring that recognition is spread equitably[6].

### Cultivate a growth mindset

One of the most important roles of a mentor is to create an environment that fosters the growth and development of the mentee and recognizes failures as important steps in the process toward success. Psychologist Carol Dweck describes two mindsets–the fixed mindset, where individuals believe their basic abilities and intelligence are fixed traits, and the growth mindset, where individuals understand that their talents and abilities can be developed through effort and persistence. A hallmark of the growth mindset is not being afraid to take risks, to seek out challenges and then learn from the results[14].

The effective mentor cultivates the growth mindset in team members, empowering them to make decisions autonomously, with a clear understanding that the mentor will be there to support and guide them if they get stuck or veer off track. In the virtual environment, particularly when communications may be limited, promoting the growth mindset can be challenging and may need to be explicitly stated by the mentor so team members feel free to take risks. Knowing that the mentor is there as a safety net enables team members to perform independently and learn from both missteps and successes. A true learning environment can flourish when the team mentor asks questions instead of providing answers, supports team members instead of judging them, and promotes their development instead of dictating to them[15].

## Stage 3: norming

In this stage of development, the virtual team is cohesive and a mentor is tasked with strengthening individual contributions to promote team function.

### Encourage peer mentorship

As a team evolves, members typically settle into roles that are comfortable for the group and enhance its productivity. In this stage, peer mentorship relationships are likely to form within the virtual team. This transformation evolves through collaboration and project-based teamwork, with support from the mentor. Peer mentorship leverages the complementary knowledge and experience of team members, who work and support each other in parallel, rather than in hierarchical relationships. Peer and near-peer team members provide valuable insight and validation to one another. [16–18] As peer mentorship relationships form on the team, mentors can more easily step back from active leadership, becoming a facilitator, role model and partner in learning.

### Share the leadership role

A mentor can promote healthy dynamics within a virtual team by engaging in shared leadership, or distributing leadership across members of the team regardless of formal role or position[19]. Moving beyond delegation, shared leadership engages the team in a truly shared sense of responsibility and purpose, and gives the mentor a chance to offer feedback on leadership skills. The literature has shown a positive correlation between shared leadership and team effectiveness, particularly in teams focused on complex tasks[20]. On virtual teams, where relationships can be more challenging to build, shared leadership has been shown to increase trust and promote team level satisfaction[21]. A mentor who takes the time to know the strengths of virtual team members and encourages a growth mindset will more easily identify appropriate tasks and individuals for sharing leadership. Fostering an environment of psychological safety will help team members step out of a comfort zone and into a leadership role. For example, a mentor could suggest that a team member lead an online meeting without the mentor present. The mentor should consider whether a pre-planning meeting is necessary to set this team member up for success.

## Stage 4: performing

In the performing stage of development, the virtual team is progressing towards its stated goals. The team mentor is charged with maintaining efficiency and productivity.

### Create structure to increase productivity

One of the biggest challenges of virtual teams is maximizing process gains such as team coordination, cooperation, and communication[22]. It is not uncommon to have turbulence as members adjust to roles and responsibilities. They begin to express their ideas and styles as they try to meet the project’s expectations while competing for a role on the team. The team mentor must meld the group together by providing structure through regular meetings with set agendas, using virtual platforms that are accessible and flexible. This structure helps to bind the team together, so members can speak openly and honestly, without getting the group off track. A team needs clearly defined goals and objectives that give each member a distinct role with clear expectations and purpose[23]. A shared leadership approach, in which roles and responsibilities are divided up based on individual strengths, augments a team’s productivity, enabling the team to realize that each member has important areas of competence to contribute. Shared leadership has demonstrated increased satisfaction on virtual teams[21]; however, a ‘gentle nudge’ by the mentor to encourage members to make this transition is often needed.

### Encourage clear communication strategies

Communication is critical when mentoring a virtual team. Collaboration at a distance relies on electronic communication, which often lacks tone and nuance, and is liable to misinterpretation[24]. Email writers are often less guarded and sometimes more negative in their electronic communications, while email recipients tend to interpret information more negatively than it is intended by the sender. In addition, important information can get overlooked because an email writer tends to overestimate the clarity of their expressed priorities[25]. An important role of the team mentor is to coach the team in communication strategies, providing explicit feedback on communication style and content in the online environment. Key strategies to role model are stating one’s intentions clearly, highlighting salient points, and reviewing one’s messages to ensure that the correct tone is conveyed[25]. In addition, because virtual teams communicate with fewer face-to-face interactions, it is difficult to gauge team members’ reactions through body language and other subtle cues. The team mentor should encourage team members to create their own ‘team charter’ to mitigate the challenges of virtual communication. This charter could include establishing standards for behavior when participating in virtual meetings, guidelines for when to send emails and when to call, and other strategies to ensure that communication between team members is clear and unambiguous [25,26].

### Mentor in the moment

As the team progresses, it begins to form a cohesive sense of purpose in which members accept each other’s roles and abilities and no longer focus on themselves, but on the tasks and overarching goals. Trust begins to grow and team members seek the insights and input of others. In this context, mentoring in the moment can be used to maximize individual performance. Team mentors should take advantage of the team’s successes and struggles, making them illustrative teaching points. Translating missteps into teachable moments can be awkward, and even threatening. Useful approaches might be: ‘I’ve noticed you working on XXX, you are doing a great job. What struggles are you having?’ or ‘I wonder if I could get your take on something I am working on. I’d value your perspective.’[27] As the discussion progresses, the mentor can bring in his or her own experiences and describe what went well and what did not[28]. Sharing personal stories and lessons learned is a non-threatening way to provide advice and guidance to the mentee. The mentor should guide mentees to solve their struggles, so they can learn from the experience. However, the mentor’s job is to balance support with challenge, so expectations must be realistic[29].

### Prompt group and individual reflection

The goal of a team is to reach a high level of performance at which everyone knows their roles and expectations and has the experience to carry them through. Reflective practice can assist in this transformative process and is generally well received by team members, leading to increased willingness to collaborate. Reflective practice helps participants understand others, recognize their niches of expertise, identify team strengths and weaknesses, appreciate the importance of working together, and value constructive conflict resolution[30]. When barriers arise, the group can handle them with ease in a collaborative manner. Teams should take time to reflect on their progress toward their goals and be comfortable enough with each other to reflect on the project from the perspective of individual roles. Initially, reflection should be conducted in an unstructured fashion, and then can proceed to a structured form with discussion of processes, actions, emotions and thoughts, relevant past experiences, and review of literature when helpful[31]. It is important that everyone is heard and clear plans are made to deal with issues at hand. Through reflection, adjustments are made to increase the team’s resiliency[32].

## Stage 5: adjourning

In this final stage of development, the team’s work is done. The mentor can role model appreciation and promote ongoing collaboration.

### Acknowledge individual contributions

As a team concludes its work, a mentor has the opportunity to help each member feel valued for their contributions. Recently, the value of appreciation has been the subject of research in economics and organization management. In one study, 81% of people indicated they would be willing to work harder if they had an appreciative manager; 70% reported they would feel better about themselves if their manager thanked them more regularly[33]. Daily appreciation has been shown to improve mindset and mood in employees, increase worker effectiveness and task performance, increase trust and loyalty among employees, and inspire more innovation in teams [34,35]. Good mentors can strengthen their team members by increasing the team members’ belief in their own ability to make a difference, even in the face of challenge[33]. A mentor can help mentees know that their opinion matters and is valued by giving them choices when making decisions to support their autonomy. Thanking mentees and team members publicly and giving them credit for their hard work is a powerful way to promote their accomplishments and ideas.

### It’s not goodbye, it’s ‘til we meet again

Throughout the course of virtual team development, the bonds created and nurtured can serve as the foundation for future endeavors. Mentors can capitalize on the relationships built and continue the collaboration as a means of enhancing personal growth and development. Team members can be a rich source of information and ideas for future projects and endeavors and collaborating from a distance enables individuals to expand their professional networks. One of the advantages of multi-site collaboration is the potential for a wider dissemination of work accomplished, which benefits all members of the virtual group and contributes to the broader medical education community[36]. Plan to present your team’s work in as many venues – local, regional, national, and international – as possible, and vary the primary speaker to provide opportunities for every team member. Become familiar with the interests and expertise of those you are working with, and look for ways to expand each other’s professional portfolios, for example as a consultant or speaker at your own institution. In fact, this article emerged from a virtual team project: the authors collaborated at a distance on an online medical education course, with mentoring of the junior team members by the senior member.

## Conclusion

Due to the pandemic, medical education teams that were accustomed to in person collaboration were forced online and technology is now at the forefront of team inner workings. Adaptations that were created by necessity have the capacity to remain relevant and change the way we continue to collaborate and mentor. Through our own personal experiences of long-distance collaboration, we have delineated mentorship strategies centered on Tuckman’s Five Stage Model of Team Development which we hope will enable teams to continue to thrive in a virtual world. Mentoring teams in the virtual environment without guidance can have its pitfalls, but with knowledge of best practices, these experiences can be leveraged to maximize team development, efficiency and production.
